# Editorial: New frontiers in radiobiology

**DOI:** 10.3389/fpubh.2023.1181656

**Published:** 2023-03-27

**Authors:** Daniel K. Ebner, Takashi Shimokawa, Walter Tinganelli, Charlot Vandevoorde

**Affiliations:** ^1^Department of Accelerator and Medical Physics, National Institutes of Quantum Science and Technology, Chiba, Japan; ^2^Department of Radiation Oncology, Mayo Clinic, Rochester, MN, United States; ^3^Biophysics Department, Helmholtz Centre for Heavy Ion Research, Darmstadt, Germany

**Keywords:** radiobiology, particle, biomarker, radioisotope, LET

The field of radiation oncology and by extension the study of radiobiology is undergoing a renaissance driven by novel synergistic treatments with chemo- and immunotherapeutic agents, novel uses of our existing technology such as in very high dose rate irradiation (FLASH), and by expanding use of particle radiotherapy for new clinical indications. In this Research Topic, jointly featured in Frontiers in Public Health and Frontiers in Oncology, we are pleased to feature nine articles that probe these novel discoveries and methodologies.

The articles highlighted here span the gamut of radiobiology. Efforts to better understand the fundamental biophysical mechanisms of charged particle radiotherapy were heavily featured. Li presented a novel analysis of the Particle Irradiation Data Ensemble (PIDE) dataset, based on 1,118 *in vitro* cell survival datasets, to present a novel model including LET and fluence of different ion species. This resulted in a model predictive of survival fraction for all ion beams, which unlike previous models is independent of reference photon irradiation and universal across all ion and cell species. Frame et al. visualized broken or intact individual DNA molecules using atomic force microscopy in order to demonstrate the increased rate of DNA breakage within the Bragg peak of a monoenergetic 110 MeV proton beam compared to the entrance plateau region. Collectively, their work on a plasmid model system reinforces the notion that a universal RBE of 1.1 for proton radiotherapy does not accurately fit the observed effects, and the role of LET in driving enhanced DNA DSB production despite delivery of the same physical dose.

The exploration continues within radiotherapeutic techniques that are less commonly used in practice today and go beyond traditional external beam radiotherapy. Wang et al. review modern boron neutron capture radiotherapy, well-delineating current status and challenges as this modality gains a modern resurgence. One of the stumbling blocks remains the targeting ability of boron delivery agents, which is directly linked to the induction of surrounding normal tissue damage. Guerra Liberal et al. extend the Topic's focus to targeted radionuclide therapy, where alpha-particle emitters are rapidly gaining momentum for the treatment of radioresistant tumors. The alpha-particle emitter, Radium-223 (^223^Ra), has been tied to improved survival in castration-resistant prostate cancer patients with bone metastasis and is administered in clinical practice in the form of ^223^Ra dichloride (^223^RaCl_2_). The authors explore the biological mechanism of its action, by comparing it to different radiation qualities such as conventional X-ray and external alpha irradiation in a range of clinically absorbed doses of 0.05–2 Gy in a panel of prostate cancer and bone cell lines. Their results uncover a delayed DNA repair between both ^223^Ra and alpha-particle modalities with similar kinetics. Collectively, they identify that the greater cell-type dependent RBE of ^223^Ra appears associated with increased levels of residual DNA damage and cell death by mitotic catastrophe. Nishri et al. explore diffusing alpha-emitter radiotherapy (DaRT) using radium-224 (^224^Ra)-loaded seeds in combination with temozolomide (TMZ) or the anti-angiogenic agent bevacizumab (BEV) in *in vitro* U87 cultured cells as well as in a glioblastoma xenograph mouse model. The results presented in this extensive preclinical study confirm that the combinational treatment with TMZ and DaRT nearly doubled cytotoxicity. This was confirmed by colony survival studies wherein the addition of TMZ reduced the surviving fraction by 40–50% compared to monotherapy. In animal studies, the combination DaRT and TMZ delayed tumor development greater than monotherapy alone, with further increased tumor control noted with BEV administration. We hope this sees successful translation to clinical trials and adds a novel therapeutic strategy to improve the survival of glioblastoma patients.

Clark et al. are seeking to understand the effects of radiotherapy on tumor-microenvironmental interactions in driving resistance transformation, by identifying extracellular vesicles produced by breast cancer cells following treatment. These vesicles promote cancer stem-like cell expansion, radioresistance and enhanced pro-tumor activity of fibroblasts *via* IL-6 production. Furthermore, they identify a potential therapeutic target as well as, in the vesicles, a potential traceable biomarker. Park et al. aim to illustrate the radioprotective potential of a bacterial exopolysaccharide isolated from *D. radiodurans* BRD125 in their work. The compound offered free-radical scavenging effects, resulting in reduced irradiation-induced apoptosis particularly within the bone-marrow and spleen of X-ray irradiated mice, combined with enhanced expression of hematopoiesis-related cytokines such as GM-CSF, G-GSF, M-CSF, and SCF, leading to enhanced hematopoietic stem cell protection and regeneration.

Certainly not least, interest in biomarker development featured prominently in our topic. The topic was interwoven in the above featured papers, as well as in the form of P53 status in Mireştean et al.s' review of the Warburg effect, where they evaluate the potential to leverage targeted “anti-Warburg” therapies to improve the radiosensitivity of head and neck cancer. Separately, Kocsis et al. aimed to evaluate a biomarker for radiosensitivity and decrease of pulmonary function, looking at chromosomal aberrations of *in vitro* irradiated blood of non-small cell lung cancer patients before the start of therapy and compare it to the aberrations in the blood of patients at several time points (ranging from 3 to 24 months) after stereotactic radiotherapy. The authors find a connection between these aberrations in the *in vitro* irradiated blood samples before the start of radiotherapy and subsequent decrease in pulmonary function tests following radiotherapy.

Radiobiology is constantly evolving, and novel treatment methodologies—as well as better understanding of our current clinical offerings—are creating new frontiers within the field. This requires an interdisciplinary approach integrating multiple scientific and clinical disciplines. This section aims to bring together a broad evaluation and overview of the current frontiers in radiobiology.

## Author contributions

All authors listed have made a substantial, direct, and intellectual contribution to the work and approved it for publication.

